# Validation of the German version of the Kujala score in patients with patellofemoral instability: a prospective multi-centre study

**DOI:** 10.1007/s00402-018-2881-5

**Published:** 2018-01-25

**Authors:** D. Dammerer, M. C. Liebensteiner, U. M. Kujala, K. Emmanuel, S. Kopf, F. Dirisamer, J. M. Giesinger

**Affiliations:** 1 Department of Orthopaedic Surgery, Medical University of Innsbruck, Anichstraße 35, 6020 Innsbruck, Austria; 2 Faculty of Sport and Health Sciences, University of Jyväskylä, Liikunta Building, P.O. Box 35 (L), FI 40014 Jyväskylä, Finland; 3 Department of Orthopaedic Surgery, Krankenhaus Barmherzige Schwestern, Seilerstätte 4, 4020 Linz, Austria; 4 Center for Musculoskeletal Surgery, Charité-University Medicine Berlin, Charitéplatz 1, 10117 Berlin, Germany; 5 Innsbruck Institute of Patient-centered Outcome Research (IIPCOR), Dr.Stumpf Straße 56, 6020 Innsbruck, Austria; 6 Orthopädie and Sportchirurgie, Karl-Leitl-Straße 1, 4048 Linz-Puchenau, Austria

**Keywords:** Kujala score, Patellofemoral instability, Validation, Kujala patellofemoral score, Patellofemoral pain syndrome (PFPS), Questionnaire

## Abstract

**Introduction:**

The Kujala score is the most frequently used questionnaire for patellofemoral disorders like pain, instability or osteoarthritis. Unfortunately, we are not aware of a validated German version of the Kujala score. The aim of our study was the translation and linguistic validation of the Kujala score in German-speaking patients with patella instability and the assessment of its measurement characteristics.

**Materials and methods:**

The German Kujala score was developed in several steps of translation. In addition to healthy controls, the Kujala German was assessed in consecutive patients undergoing reconstruction of the medial patellofemoral ligament for recurrent patellar dislocations. Pre-op, 6 and 12 months postop the patients completed the Kujala German score, the KOOS, the Lysholm score, a VAS Pain, and the SF-12v2 scores. In addition, there was a Kujala German Score retest preop after a 1-week interval.

**Results:**

We found high reliability in terms of internal consistency for the Kujala score (Cronbach’s alpha = 0.87). Convergent validity with the KOOS (symptom *r* = 0.65, pain *r* = 0.78, ADL *r* = 0.74, sports/recreation *r* = 0.84, quality of life *r* = 0.70), the Lysholm score (*r* = 0.88) and the SF-12 physical component summary score (*r* = 0.79) and VAS pain (*r* = − 0.71) was also very high. Discriminant validity in terms of correlation with the SF-12 mental component summary Score was satisfactory (*r* = 0.14).

**Conclusions:**

In conclusion, the German version of the Kujala score proved to be a reliable and valid instrument in the setting of a typical patellofemoral disease treated with a standard patellofemoral procedure.

## Introduction

The field of patellofemoral disorders and accordant therapeutic interventions is a high-turnover research field. For variable patellofemoral disorders many new therapeutic concepts have been published recently (e.g. reconstruction of the medial patellofemoral ligament [1, 2], arthroscopic trochleoplasty [3], medial patellotibial ligament reconstruction [4], matrix-associated autologous chondrocyte implantation [5]). To assess the clinical outcome of emerging techniques reliable and valid measurement instruments are of utmost importance.

In this regard, Watson et al. [6] criticised that many knee scores are used for patellofemoral disorders although they were originally not designed for that particular population. Although there are plenty of knee scores, only some of them were found to be appropriate for the field of patellofemoral disorders. Watson et al. [7] reported high test reliability and moderate responsiveness for the Kujala score. Also Crossley et al. [8] tested several outcome measures used in patients with patellofemoral disorders and recommended the Kujala score from their findings concerning validity, reliability and responsiveness.

This is reflected by today’s frequent use of this instrument by researchers. For example, when screening the literature for outcome measures used by studies investigating trochleoplasty (a surgical procedure used in the field of patellofemoral disorders), it becomes evident that 72% of the studies applied the Kujala score [9–26].

To date, the Kujala score has been validated in a number of languages [27–32], but not in German. The Kujala score was certainly used in German-speaking patients [21, 26, 33–35], but an official German version that was translated and linguistically validated in line with the recommendations of pertinent guidelines [36] as well psychometrically validated is still lacking.

Therefore, the aim of our study was the translation and linguistic validation of the Kujala score in German-speaking patients and the assessment of its measurement characteristics. In detail, we evaluated the following psychometric aspects of the German-language version of the Kujala score as recommended by the COSMIN guidelines [37]:

- Dimensionality (internal consistency)
- Test–retest reliability
- Construct validity (convergent, discriminant and known-groups validity)
- Responsiveness (pre-surgery, 6 and 12 month follow-up)
- Missing responses and floor/ceiling effects

## Materials and methods

### Translation procedure

Translation of the Kujala score into German followed the ISPOR guideline for good translation practice [36]. Three translators native in German independently translated the English version of the Kujala score [7] into German. The three translations were then harmonised in an expert panel including a professional translator for English, a specialist in cross-cultural adaptation and two knee surgeons to ensure understandability and cross-cultural equivalence of the questionnaire content. The resulting version was translated back into English by a bilingual native German speaker—blinded to the original English version—and then compared with the English-language original by the developer of the score to confirm that the meaning of the original questionnaire was maintained. If deemed necessary, adaptations were proposed to the expert panel and discussed. The German version was then discussed with ten patients to identify possible problems with the translation. Patient feedback gave no cause to make changes.

## Sample and assessment procedure

### Patients

To test the above-mentioned study aims, a prospective design was applied. Before commencement of the study the protocol was approved by the Ethics Committee. To validate the Kujala score, we administered it to German-speaking patients who underwent surgery for patellofemoral instability (medial patellofemoral ligament reconstruction with facultative concomitant techniques). The surgical procedures were performed as part of the clinical routine of the participating centres. Patients were recruited for the study in line with the following enrolment criteria:

- No other previous or current knee disorder beyond patellar instability
- Fluency in German
- Written informed consent

### Healthy controls

For known-group comparisons we also recruited an age- and sex-matched sample of healthy controls (two controls per patient). This was done via a mail survey that included study information, the questionnaires and a pre-paid envelope.

### Assessment procedure

Patients completed the Kujala score twice preoperatively (1-week interval in between) and at 6 and 12 months postoperatively. In addition to the Kujala score, patients also completed the following other questionnaires (preoperatively, 6 and 12 months postoperatively): knee injury and osteoarthritis outcome score (KOOS), the Lysholm score, a visual analogue scale (VAS) for pain and the SF-12. Healthy controls completed the Kujala score in one single sitting.

## Outcome measures

### Kujala score

The Kujala score is a 13-item questionnaire for the patient-reported assessment of anterior knee pain [7]. The score was originally introduced for patients with a variety of patellofemoral disorders and tested by the developer in the cohorts ‘anterior knee pain’, ‘patella dislocation’, ‘patella subluxation’ and healthy controls. The Kujala score asks about the ability to do several activities (squatting, stair climbing, running) and also the presence of symptoms/disabilities as noticed by the patient (limping, thigh atrophy, swelling, etc.). The items are summed up to give a total score ranging from 0 to 100, with high scores indicating good outcome. The developer reported average values of 99.9 for healthy controls, 82.8 for patients with anterior knee pain and 62.2 for patients with patella instability. The Kujala score is the most frequently used patient-reported outcome measurement in patients with patellofemoral disorders with high reliability and validity reported for the original English-language version [6, 8].

### Knee injury and osteoarthritis outcome score (KOOS)

The Knee injury and osteoarthritis outcome score (KOOS) [38] is a well-validated general knee score, developed for the assessment of sports injuries and outcomes in young and middle-aged individuals [39] and has been validated in German [40]. The KOOS consists of five subscales: pain, symptoms, activities of daily living, sports and recreation function and knee-related quality of life. Each scale ranges from 0 to 100 with high scores indicating good outcome.

### Lysholm score

The Lysholm score is another well-established knee outcome score with a specific focus on knee ligament surgery [41]. A German-language version of the score was recently validated [42].

### Pain scale

A rating of usual knee pain in the last 7 days was obtained from all patients using a 0–100 mm visual analogue scale (VAS).

### Short-Form 12

The Short-Form 12 (version 2, German; SF-12v2) [43] was included in the study as a measure of general health to complement the joint-specific questionnaires. The SF-12 consists of 12 items that are aggregated to a mental- and a physical component summary score. High scores indicate good health.

### Data analysis

To investigate the dimensionality of the German Kujala score, we calculated Cronbach’s alpha and item-total correlations. In addition, we conducted an exploratory principal component factor analysis and investigated the eigenvalues and explained variance of the extracted factors.

To assess retest reliability we assessed patients twice before surgery after a time interval of 1 week. An intra-class correlation coefficient (ICC) was calculated and we considered a coefficient above 0.70 to indicate high retest reliability.

Convergent validity of the Kujala score was assessed in terms of Spearman rank correlation with the KOOS, the Lysholm score, the VAS pain scale and the SF-12 Physical component score. Discriminant validity was investigated using the correlation with the SF-12 mental component score. Correlation coefficients of *r* > 0.50 were deemed to indicate convergent validity and correlations of *r* < 0.35 discriminant validity [44]. For determination of known-groups validity we compared the healthy controls and the patient sample in a Mann–Whitney *U* test.

To evaluate missing responses we determined the response rate per item. To assess floor and ceiling effects, we calculated the percentage of patients obtaining the lowest or highest possible score on the Kujala total score at each assessment. A percentage of 15% was deemed to indicate substantial floor or ceiling effects [45].

To assess responsiveness of the Kujala score we calculated change over time for pre-surgery to 6 months and for pre-surgery to 12 months. We calculated the effect size Cohen’s d as a measure of change over time.

Our study was powered to detect a minimally important change (MIC) in the Kujala score between two time points. Based on the literature, we determined the MIC to be 10 points [8] and the standard deviation to be 19 points [29], giving an effect size of Cohen’s *d* = 0.53 to be detected. This effect size corresponds to general recommendations on MICs in the literature [46]. A sample of 30 patients provides 80% power to detect a change between two time points with an effect size of Cohen’s *d* = 0.53 in a *T* test for dependent samples (two-sided, alpha = 0.05).

With regard to retest reliability a sample size of 30 patients is sufficient to show that *r* > 0.70, the threshold for good retest reliability [47] provided the observed correlation coefficient is at least 0.87 (alpha = 0.05; power = 0.05; one-sided), a value frequently exceeded in previous validation studies [28].

For analysis of convergent validity a sample of 30 patients permits us to demonstrate that *r* > 0.50 if the observed correlation exceeds 0.77 (alpha = 80; power = 0.80; one-sided).

## Results

### Sample characteristics

A total of 30 patients were recruited at the participating centres. Mean patient age was 24.0 years (SD 8.1) and 76.7% were female. Side of the operated knee showed an equal split. All patients completed the retest assessment prior to surgery; 29 patients completed the questionnaire at the 6-month follow-up and 22 patients at the 12-month follow-up. For details, see Table 1.

**Table 1:** Sample characteristics for patients and healthy controls

	Patients *N* = 30		Healthy controls *N* = 60	
Age				
Mean (SD)	24.0 (8.1)		27.9 (13.8)	
Range	14–42		11–60	
Sex				
Women	76.7%	*N* = 23	75.0%%	*N* = 45
Men	23.3%	*N* = 7	25.0%%	*N* = 15
Side				
Left	50.0%	*N* = 15		
Right	50.0%	*N* = 15		
Type of surgery				
MPFL	80.0%	*N* = 24		
MPFL + trochleoplasty	10.0%	*N* = 3		
MPFL revision	3.3%	*N* = 1		
MPFL + derotat. femur	3.3%	*N* = 1		
MPFL + MACI	3.3%	*N* = 1		

*MPFL* medial patellofemoral ligament, *MACI* matrix-associated autologous chondrocyte implantation, *SD* standard deviation

The healthy controls (*n* = 60) had a mean age of 27.9 years (SD 13.8) and 75.0% were female.

No statistically significant correlation between pre-surgery Kujala score and age was observed in the patient sample (*r* = − 0.18; *p* = 0.390). Men and women were also not seen to differ significantly (74 vs 61 points; *p* = 0.220).

### Dimensionality and retest reliability

Cronbach’s alpha for the Kujala score was 0.87 with no item substantially lowering this value (exclusion of Item 11 increased Crohnbach’s alpha to 0.88). Corrected item-total correlations were lowest for Item 12 (0.32), Item 11 (0.39) and Item 10 (0.41), and highest for Item 1 (0.75) and Item 6 (0.76).

The exploratory principal component factor analysis resulted in two factors with an eigenvalue above 1.0 (factor 1: 6.1; factor 2: 1.2). The first factor explained 46.7% of variance and the second factor 9.2%. Applying Varimax rotation to a two-factor structure showed that Items 4, 7, 10, 12, 13 load strongest on the second factor. Retest reliability was found to be high with an ICC of 0.93.

### Construct validity

Correlations with the KOOS subscales, the Lysholm score and the SF-12 physical component score were all above *r* = 0.65 (KOOS symptoms subscale). Highest correlations were found with the Lysholm score (*r* = 0.88), the KOOS Sports/recreation subscale (*r* = 0.84) and the SF-12 physical component score (*r* = 0.79). Correlation with the SF-12 mental component score was *r* = 0.14, indicating good discriminant validity. For further details see, Table 2.

**Table 2:** Convergent and discriminant validity of the Kujala Score

	Correlation	*p* value
KOOS symptoms	0.65	< 0.001
KOOS pain	0.78	< 0.001
KOOS ADL	0.74	< 0.001
KOOS recreation/sports	0.84	< 0.001
KOOS QOL	0.70	< 0.001
Lysholm score	0.88	< 0.001
VAS pain	-0.71	< 0.001
SF-12 physical component score	0.79	< 0.001
SF-12 mental component score	0.14	0.279

Comparison of the patient sample at baseline as compared to the healthy controls showed a statistically significant difference for the Kujala score (*p* < 0.001), with a mean patient score of 64.5 (SD 18.0) versus a mean score of 96.5 (SD 6.4) in the healthy controls. At 12 months the difference was still statistically significant (*p* < 0.001), with patients scoring on average 81.9 (SD 18.4).

### Responsiveness

To assess responsiveness over time, we compared effect sizes for change from pre-surgery to 6 months and from pre-surgery to 12 months between the various outcome measures.

For change from pre-surgery to 6 months the effect size for the Kujala score was Cohen’s *d* = 0.57, and for 12 months it was *d* = 0.96. For early follow-up, the largest effect sizes were observed for the KOOS quality of life subscale (*d* = 1.08) and the Lysholm score (*d* = 0.93). Least change was found for KOOS symptoms (*d* = 0.04) and KOOS pain (*d* = 0.31). For late follow-up change was most pronounced for the KOOS quality of life subscale (*d* = 1.56) and the SF-12 physical component score (*d* = 1.27). KOOS symptoms (*d* = 0.38) and KOOS pain (*d* = 0.57) again showed the least change. For further details see, Table 3.

**Table 3:** Responsiveness of the Kujala score and the comparator measures

	Pre-surgery		6 months		12 months		Effect sizes for change over time	
	Mean	SD	Mean	SD	Mean	SD	Pre-surgery to 6 months	Pre-surgery to 12 months
Kujala score	64.5	18.0	74.9	19.6	81.9	18.5	0.57	0.96
KOOS symptoms	69.8	16.5	70.5	17.3	76.1	18.5	0.04	0.38
KOOS pain	68.1	21.6	74.7	18.2	80.4	15.6	0.31	0.57
KOOS ADL	75.4	18.9	83.7	14.7	87.1	17.5	0.44	0.62
KOOS recreation/sports	39.5	32.1	58.1	26.9	67.9	24.1	0.58	0.88
KOOS QOL	35.3	16.9	53.6	24.4	61.6	25.3	1.08	1.56
Lysholm score	59.4	20.7	78.6	17.5	81.2	18.7	0.93	1.05
VAS pain	5.2	2.9	2.6	2.5	2.4	2.4	− 0.88	− 0.96
SF-12 physical component	39.8	7.5	45.3	9.0	49.3	7.1	0.74	1.27

### Floor and ceiling effects and missing responses

**Fig. 1:**
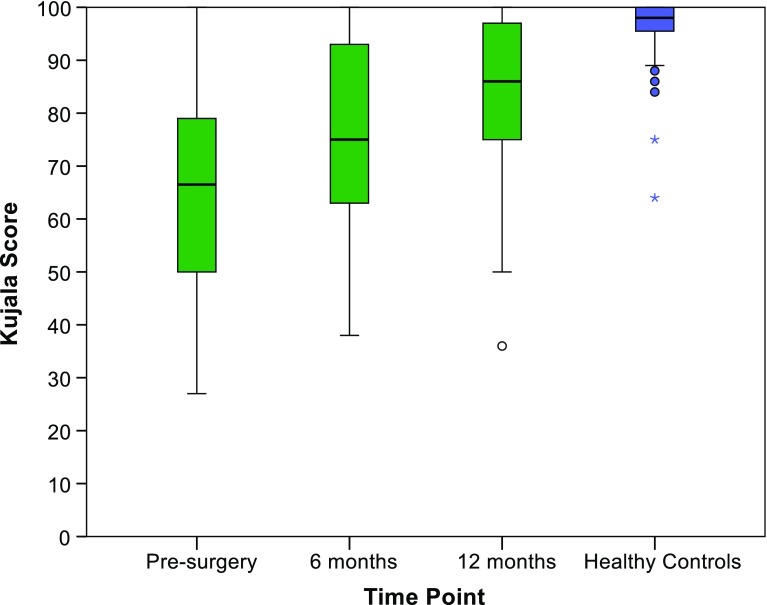
Kujala score at pre-surgery, 6 and 12 months and in the healthy controls

All patients answered all Kujala questions at each of the three study time points (pre-surgery, 6 and 12 months postoperative). The ceiling effect of the Kujala score increased over time, with 3.3% of the patients obtaining the best possible score pre-surgery, 6.9% at 6 months, and 13.6% at 12 months. No patient achieved the worst possible score at any time point. Details are shown in Fig. 1.

## Discussion

Our study demonstrated high internal consistency of the German-language version of the Kujala score in terms of Cronbach’s alpha (0.87) despite the findings of the exploratory factor analysis that showed a possible second factor consisting of Items 10 (swelling), 11 (abnormal painful kneecap movements) and 12 (thigh atrophy). Retest reliability over a 1 week preoperative period was also high (0.93). Correlations with the German-language versions of the KOOS, the Lysholm score, the SF-12 physical component score and a VAS pain scale exceeded our predefined threshold for good convergent validity of *r* = 0.50. The weak association of *r* = 0.14 with the SF-12 mental component was well below the threshold for discriminant validity of *r* = 0.35. Analysis of responsiveness to change from pre-surgery to 6 and 12 months showed that the magnitude of change of the Kujala score ranked in the middle when compared against the five KOOS subscales, the Lysholm Score, the VAS Pain and the SF-12 physical component score. The Kujala German discriminated well between healthy controls and patients pre-surgery as well as at 12-month follow-up. We did not observe a relevant floor or ceiling effect for the Kujala Score at any of the study time points, with the highest percentage (13.6%) of extreme scores being at the upper end of the scale at the 12-month follow-up.

We compared our findings with the findings previously made for the original English-language version of the Kujala score. The developers of the score neither compared the score with other scores, nor did they perform reliability tests [7]. However, they reported that the score significantly differed between different clinical populations (anterior knee pain, patella dislocation, patella subluxation, controls). The authors further verified correlations between the Kujala score and several variables of radiographic patellofemoral tracking.

We then attempted to compare the findings from the current study with those from the original English-language version of the Kujala score as made by centers other than the developer’s center. In this regard our observations were congruent with those made by Watson et al. [6], who also found high reliability and moderate responsiveness of the English version of the Kujala score. Our findings are partly congruent with those of Crossley et al. [8], who reported high reliability but also high responsiveness. They also stated that the English version of the Kujala score showed high validity and consequently recommended that the Kujala score be used in patients with patellofemoral disorders.

The Kujala score is certainly the most commonly used patient-reported outcome measurement in the field of patellofemoral disorders. This is documented by the fact that data pooling during meta-analysis in the field of patellofemoral disorders is typically done with the Kujala score [48]. The importance of the Kujala score is shown by the fact that it was previously translated to Spanish, Chinese, Dutch, Greek, Thai, Turkish, Persian and Brazilian Portuguese [27–32, 49, 50]. Regarding internal consistency, all those publications reported Cronbach’s alpha values of around or above 0.8 (except for the Thai version of the Kujala score, whose internal consistency was not reported). All the above-mentioned papers investigated the test–retest reliability and reported good to excellent results. However, some of the previous researchers applied very short test–retest intervals (30 min, 1–2 days) [30, 32]. Except for the Dutch, Turkish and Thai Kujala versions, the above-mentioned studies also examined the construct validity of the respective Kujala score versions in comparison to that of other typical questionnaires (VISA-P, WOMAC, SF-36). Similar to the findings of the current study, good results were reported. Another consistent finding across the above-mentioned studies including our own study is the lack of floor/ceiling effects of the Kujala score, which makes it a useful assessment tool at late follow-up time points when symptoms have mostly recovered and patient burden is low. However, floor/ceiling effects were not investigated for the Dutch, Thai, Chinese and Turkish Kujala versions. Except for the Spanish version [28], none of the above-mentioned studies investigated the responsiveness of the particular score version. For the Spanish version good responsiveness was reported.

More detailed analyses of dimensionality using, e.g. item response theory techniques or confirmatory factor analysis are currently not available in the literature. Given the exploratory results for the factor structure of the Kujala score in our study, this may be a worthwhile focus of research in future studies.

A limitation of our study was the sample size that did not allow us to conduct more detailed subgroup analyses. Furthermore, the healthy control group consisted of a convenience sample. Therefore, the control group was not representative of the general population and was somewhat older than the patients. Strengths of our study are comprehensive analyses of various measurement characteristics of the Kujala score and the longitudinal design of our study. Another strength is that, in contrast to other studies that translated the Kujala score to several languages (other than German), the population of the current study was very homogeneous (medial patellofemoral ligament reconstruction surgery in the setting of patellofemoral instability).

The study findings are deemed to be of high clinical relevance in light of the fact that the Kujala score is the most common questionnaire applied in the field of patellofemoral disorders. The now available German-language version should facilitate clinical and scientific work of high quality in the field of patellofemoral disorders.

In conclusion, the German version of the Kujala score proved to be a reliable and valid instrument in the setting of a typical patellofemoral disease treated with a standard patellofemoral procedure. As a condition-specific score it can be used in combination with general health questionnaires to obtain comprehensive information on treatment impact from the patient perspective.

## Abbreviations

KOOS: Knee injury and osteoarthritis outcome score
VAS: Visual analogue scale
SD: Standard deviation
PFPS: Patellofemoral pain syndrom
COSMIN: Consensus-based standards for the selective of health measurements instruments
ISPOR: International society for pharmacoeconomics and outcome research
SF: Short form
ICC: Intra-class correlation coefficient
MIC: Minimally important change
WOMAC: Western Ontario and McMaster Universities arthritis index
